# Primary Mandibular Tuberculous Osteomyelitis Mimicking Ameloblastoma: A Case Report and Literature Review of Mandibular Tuberculous Osteomyelitis

**DOI:** 10.1055/a-2217-8784

**Published:** 2024-02-29

**Authors:** Chandrashekhar Chalwade, Armaan Khosa, Kishor Ballary, Raghav Mago

**Affiliations:** 1 Department of Plastic Surgery, KEM Hospital, Parel, Mumbai, Maharashtra, India; 2 Plastic Surgery Clinic, Nayak Solitaire, Hosur, Hubli, Karnataka, India

**Keywords:** mandible, osteomyelitis, tuberculosis

## Abstract

Primary tuberculous osteomyelitis involving the mandible represents less than 2% of skeletal locations. In this paper, we report a case of mandibular tuberculosis (TB) detected after histopathological analysis of the surgically resected specimen during surgical management of a suspected case of ameloblastoma. A 14-year-old male patient presented to us with history of right-sided chin swelling. The clinical examination revealed a swelling, involving right body and parasymphysis of mandible, measuring approximately 6 cm in length and 2 cm in width, extending from right lateral incisor till the first molar. Radiological scans revealed a large multiloculated osteolytic expansive lesion measuring 52 × 20 × 18 mm. Excision of the lesion was performed and reconstruction was done with iliac bone grafting. The histopathological findings revealed a granulomatous lesion, suggestive of tuberculous osteomyelitis. The patient was successfully treated with standard multidrug therapy. One year after completion of therapy, there were no signs of recurrence. Primary mandibular TB is an extremely rare entity. Its clinical presentation is not specific. Radiologically, TB has no characteristic appearance. The positive diagnosis is based on histology. Primary mandibular TB is rare and should be kept among differential diagnoses in susceptible population and in endemic areas.

## Introduction

Primary tuberculosis (TB) of the mandible is rare. It represents less than 2% of the skeletal locations. 1 2 3 TB of the mandible usually presents as a part of multifocal lesions in the body, involving other bones and lungs. 4 A total of 1.6 million people died from TB in 2021 (including 187,000 people with HIV). In 2021, an estimated 10.6 million people were infected with TB worldwide. 5 Mandibular TB is uncommon and often diagnosed late because of nonspecific clinical presentation and the absence of pathognomonic signs. 6 7 The mainstay of treatment is antitubercular multidrug therapy. Surgery is indicated in only some cases. 8 9 We report the case of a 14-year-old male patient managed for mandibular swelling, with provisional diagnosis of benign mandibular etiology, likely ameloblastoma. Primary mandibular TB was detected incidentally on the histopathological examination. 10

## Case

**Fig. 1:**
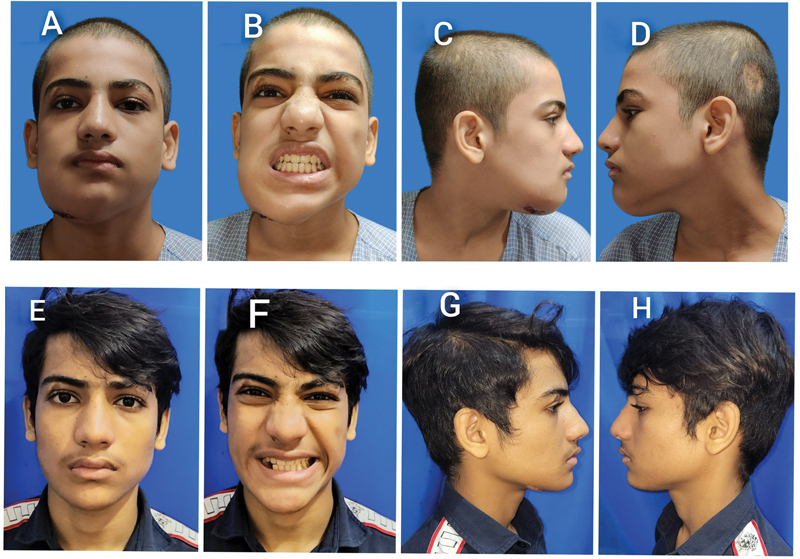
Pre- and postoperative pictures of facial profile of patient. ( **A** ) Preoperative frontal view. ( **B** ) Preoperative occlusion. ( **C** ) Preoperative right lateral view. ( **D** ) Preoperative left lateral view. ( **E** ) Postoperative frontal view. ( **F** ) Postoperative occlusion. ( **G** ) Postoperative right lateral view. ( **H** ) Postoperative left lateral view.

A 14-year-old male patient presented to us with a history of painless, gradually increasing swelling on the right side of chin for 1 year ( Fig. 1A–D ). He did not have any history of trauma, discharge, sinus, or redness in the region or any sudden increase in size. There was a scar of previous biopsies over the right side of his chin. There was no history of TB or family history of any chronic disease. Clinical examination revealed a swelling involving right parasymphysis and body of mandible, measuring approximately 6 cm in length and 2 cm in width, extending from right lateral incisor till the first molar, with normal overlying skin. On palpation, the swelling was firm in consistency. It was noncompressible, nonfluctuant, and slightly tender. The oral examination of the patient showed good mouth opening with normal occlusion and fair hygiene. His canine and first premolar over the involved bone were loose. There were no palpable lymph nodes. Systemic examination was normal.

**Fig. 2:**
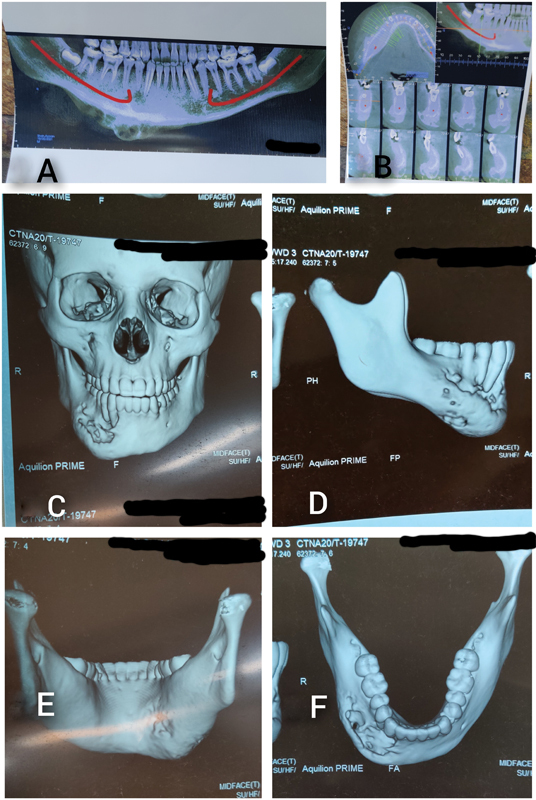
Radiological investigations. ( **A** ) Preoperative orthopantomogram showing the multilocular osteolytic lesion of right parasymphysis and body of mandible. ( **B** ) Cone beam CT showing the lytic lesion involving right side of the mandible from lateral incisor till first molar. ( **C** ) CT three-dimensional reconstruction showing multiloculated osteolytic expansive lesion arising from the right parasymphysis and body of mandible. ( **D** ) CT plate showing involvement of anterior cortex of mandible. ( **E** ) CT plate showing sparing of posterior cortex of mandible. ( **F** ) CT plate showing involvement of right side of the mandible from lateral incisor till first molar. CT, computed tomography.

The blood investigations and serology were within normal limits. The orthopantomogram ( Fig. 2A ) showed a multilocular osteolytic lesion involving the right parasymphysis and body of the mandible. Computed tomography (CT) scan ( Fig. 2B–F ) revealed a large multiloculated osteolytic expansive lesion arising from the right parasymphysis and body of mandible measuring 52 × 20 × 18 mm. The posterior cortex was not involved by the disease process. No enlarged lymph nodes were identified. The patient had undergone biopsies of the lesion at two different centers on separate occasions. These biopsies did not show any cellular atypia. However, they were inconclusive for any definitive ethology. Chest X-ray was found to be normal. Positron emission tomography scan was done—lytic lesion is noted in body of right mandible with soft tissue and fat stranding—there is significant cortical breech and minimal periosteal reaction known as osteomyelitis involvement. Few tiny, right-sided, cervical level IB, Il and Bilateral level V nodes are noted and appeared reactive. Enlarged prevascular lymph node is noted measuring 2.0 × 1.5 cm; similar left-sided hilar node is also noted. Rest of the bones were visualized and bilateral lungs appeared unremarkable. Remainder of the survey showed unremarkable tracer distribution. Our provisional diagnosis was benign mandibular ethology, likely ameloblastoma.

**Fig. 3:**
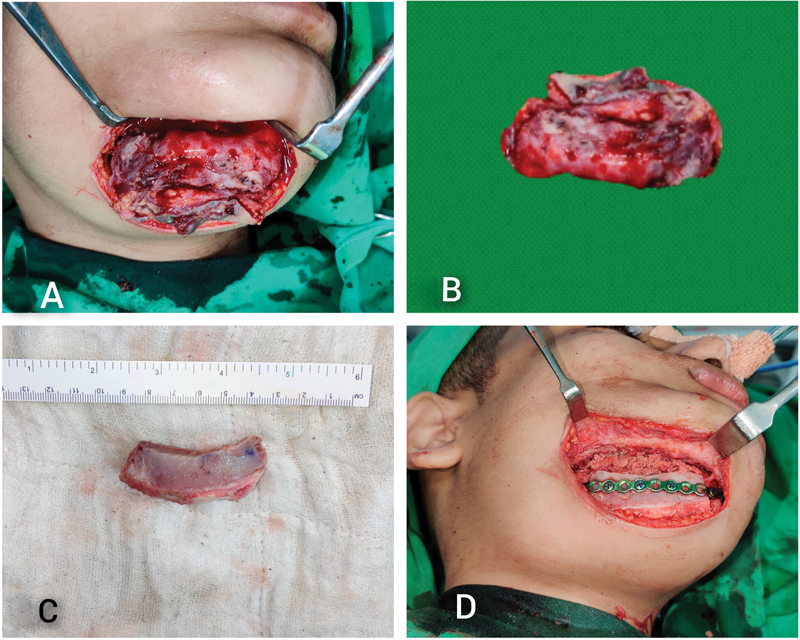
Intraoperative pictures. ( **A** ) External approach for the lesion. ( **B** ) Surgically resected specimen measuring 5 × 2 × 1.8 cm. ( **C** ) Anterior surface of corticocancellous iliac crest bone graft measuring 5.5 × 2 cm. ( **D** ) Single 2.5-mm continuous miniplate in situ for fixation of bone graft.

Informed consent was taken from the patient and his father for publication. The surgical management involved excision of the lesion with external approach ( Fig. 3A ). The specimen was greyish, gritty, and with destruction of the anterior cortex of the mandible. The canine and first premolar were involved. The specimen measurements were 5 × 2 × 1.8 cm ( Fig. 3B ). Corticocancellous iliac crest bone graft, measuring 5.5 × 2 cm was used for reconstruction of the defect ( Fig. 3C ). A single, continuous, 2.5-mm miniplate was used for fixation of bone graft using 2.5-mm screws ( Fig. 3D ). The postoperative period was uneventful.

The histopathological analysis of the excised specimen revealed a granulomatous lesion, consistent with tuberculous osteomyelitis. On gross examination, a 1.6 × 1.2-cm cystic cavity was seen at one extreme, lined by necrotic material. On microscopy, multiple sections showed epithelioid granulomas, giant cells, and caseous necrosis. Necrotic bone was noted. Patient was started on category I of antituberculosis therapy (ATT) as per The National Tuberculosis Elimination Programme regimen. It included intensive phase therapy for 2 months, consisting of rifampicin, isoniazid, pyrazinamide, and ethambutol, followed by rifampicin, isoniazid, and ethambutol as continuation phase therapy for 4 months.

**Fig. 4:**
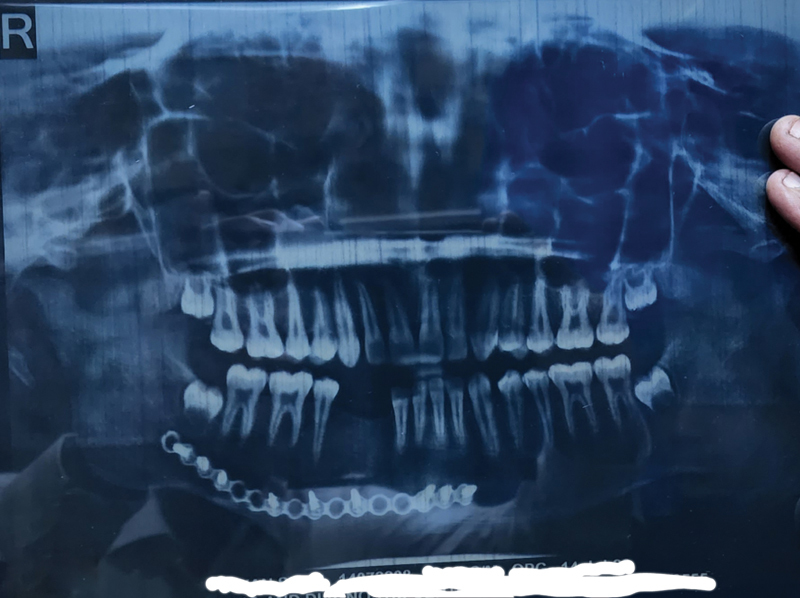
Postoperative OPG. Postoperative OPG showing integration of bone graft and miniplate with screws in situ. OPG, orthopantomogram.

On follow-up ( Fig. 1E–H ) at 1 year after completion of ATT (18 months after surgery), the patient had no complaints and no evidence of recurrence. He has good mouth opening, normal occlusion, and good aesthetic outcome. Radiological evaluation ( Fig. 4 ) showed integration of bone graft. Patient is now being worked up for dental rehabilitation.

## Discussion

Ameloblastoma is a benign odontogenic tumor. 11 It is usually asymptomatic but when it attains considerable size, it can present with jaw expansion. Radiologically, it shows an osteolytic pattern. Approximately 80% of these tumors are found in the mandible, but the maxilla is infrequently affected. 12

Oral TB may manifest as swelling, pain, loosening of teeth, displacement of tooth buds, ulcers, granulomas, involvement of salivary glands and temporomandibular joint, and tuberculous lymphadenitis. 13 The lesions of primary orofacial TB could be the only presentation of the disease. 14 Primary TB of the mandible is an uncommon entity which represents less than 2% of all skeletal TB. 1 2 3 As the mandible contains less cancellous bone, the chances of involvement of the mandible are very less in comparison to maxilla, except that the alveolar and angle regions have greater affinity. 15 In orofacial region, the mandible is affected more often than the maxilla and the angle and alveolus of the mandible are commonly affected areas. 7

Different mechanisms of extension of the infection to the mandible involve direct inoculation through dental extraction or lesions of mucosa or perforation of an erupting tooth, extension from a nearby soft tissue lesion which involves the underlying bone and hematogenous seeding. 16 17 Mandibular TB is often difficult to diagnose because of the uncommon and nonspecific presentation and absence of pathognomonic signs. In a few cases, it appears as an acute inflammatory swelling. 7 In countries like India, TB can never be left out of the differential diagnoses. Elzouiti et al reported a patient of ameloblastoma whose mandibular TB was diagnosed incidentally on histopathology, which is extremely rare. 6

Radiologically, there is no characteristic appearance of TB. 16 Mandibular TB starts as an area of rarefaction and trabecular blurring. Slowly, there is erosion of cortical bone and it is replaced by soft granulation tissue and subsequently subperiosteal abscess formation takes place ending with a visible painful swelling. Pathological fractures of the mandible or sequestration have also been reported. 18

The cases with minimally destructive lesions of TB can be managed by ATT. The medical treatment includes rifampicin, isoniazid, pyrazinamide, and ethambutol initially as an intensive phase regimen followed by rifampicin, isoniazid, and ethambutol as continuation phase for a total period of 6 to 18 months with clinical and biological monitoring. 19 Surgical modalities described are for abscess developing in the soft tissue and removal of the sequestered necrotic bone. Decortication of bone is indicated for moderately destructive lesions because of medullary bone destruction and/or cortical bone perforation. 2 Early detection of the disease results in complete cure and can lead to reversal of all destructive bony changes. If detected late, this can lead to serious complications like tuberculous meningitis. 4

Difficulty in differentiating primary tuberculous osteomyelitis of mandible from ameloblastoma stems from the fact that both can present as slowly growing expansile osteolytic lesion in the mandible. Discharging sinuses and signs of TB elsewhere in body may point to tuberculous osteomyelitis. But as in our case, primary tuberculous osteomyelitis of mandible may present without any of these. Bone sequestration, abscess formation are common in tuberculous involvement, however these are not pathognomic and can rarely be found in ameloblastoma. Definitive diagnosis in absence of systemic findings of TB rests on histopathological examination.

**Fig. 5:**
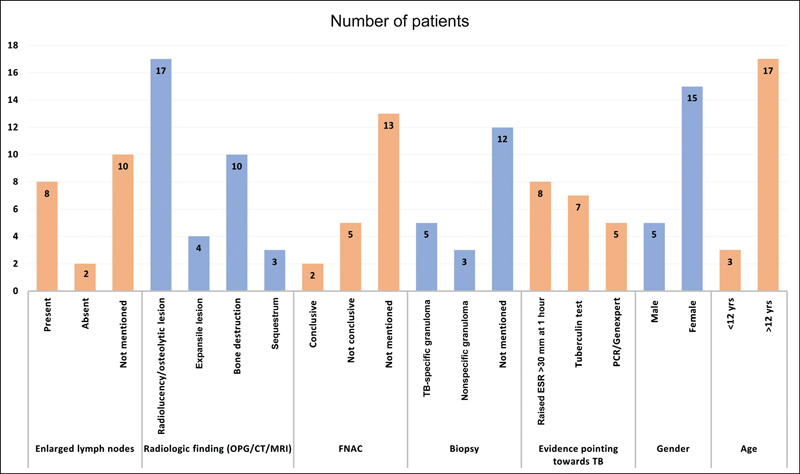
Comparative chart showing the number of patients with various findings in terms of enlarged lymph nodes, radiological finding, FNAC, biopsy, evidence pointing towards TB, gender and age of the patient. ESR, erythrocyte sedimentation rate; TB, tuberculosis; yrs, years; FNAC, fine needle aspiration cytology.

We have carried out a review of 20 cases of primary TB of the mandible ( Fig. 5 , Table 1 ) reported in literature. 3 6 14 15 16 20 21 22 23 24 25 26 27 28 29 30 31 32 33 34 We excluded all the cases which had signs of secondary involvement of the mandible and included cases which had no signs suggestive of TB anywhere else in the body. Although tuberculous osteomyelitis is more prevalent in endemic areas, it is present all over the world and among all age groups. It usually begins as a localized swelling and is associated with pain and enlarged cervical lymph nodes. While cytological examination is mostly inconclusive, biopsy could provide the necessary information to diagnose tuberculous osteomyelitis. However, biopsy is not always conclusive for TB, like in our scenario. Radiological imaging with findings like radiolucency, bone destruction, osteolytic and expansile bone lesions or sequestrum should raise the suspicion of osteomyelitis. In cases with raised erythrocyte sedimentation rate and positive tuberculin test, along with findings of osteomyelitis on imaging and pathological examination, one should always rule out TB in endemic regions. We observed that ATT was sufficient for complete resolution of the symptoms in most of the studies. Surgery was done in cases where there was diagnostic uncertainty.

**Table 1:** Analysis of reviewed case reports and studies

Publication (author/year)	Patient demography age/sex	Main presenting symptom Swelling (Location/Tender/Duration)	Enlarged neck lymph nodes	FNAC (conclusive y/n)/na	Biopsy (specific/nonspecific granuloma)	OPG/CT/MRI (radiolucent-osteolytic/expansile/bone destruction/sequestrum)	Other investigations/Evidence before surgery in pointing towards TB	Management	Final HPE	Follow-up/recurrence	Comment
Nwoku et al, 1983, Nigeria 20	8-year-old female	LJ/y/4 years	y	na	y/n	y/na/y/na	–	ATT	–	Complete resolution	–
Sepheriadou-Mavropoulou and Yannoulopoulos 1986, Greece 21	35-year-old male	RJ/y/na	na	na	na	y/na/na/na	Chest X-ray, routine lab parameters normal	Curetting, removal of sequestra, ATT after HPE diagnosis	F/s/o Tuberculous osteomyelitis	Complete resolution, no recurrence	–
Fukuda et al, 1992, Japan 22	76-year-old female	LJ/y/1 week	y	na	na	y/na/y/na	ESR = 80 mm/hr, tuberculin test positive	Saucerization done ATT given	Destruction of bone, marrow fibrosis. Connective tissue with Langerhans giant cells, epithelioid cells	No recurrence	–
Soman and Davies, 2003, United Kingdom 23	37-year-old female	LPA/na/na	na	na	na	n/na/na/na	Sputum neg, ESR = 37 mm/hr, Montoux positive, Heaf test strong grade 3 reaction	TMJ exploration with shave of condyle ATT after HPE	F/s/o tuberculous	Resolution	–
Modali et al, 2003, India 24	19-year-old female	LJ/y/na	na	na	na	y/na/na/na	ESR = 50 mm/hr, AFB neg, Tuberculin test neg	Patient underwent curettage first, then hemimandibulectomy at 1 year ATT was started after the second procedure	F/s/o tuberculous osteomyelitis	Recurrence after first procedure Resolution after second procedure and starting of ATT No recurrence after that	Patient was probably uncooperative and was not following up as advised
Dinkar and Prabhudessai, 2008, India 16	10-year-old female	LJ/y/2 months	n	y	y/n	y/na/na/na	ESR = 22 mm/hr, tuberculin test neg	ATT	–	Complete resolution	–
Helbling et al, 2010, Switzerland 25	22-year-old female	LPA/y	na	na	n/y	y/y/y/na	Sputum AFB neg, chest X-ray normal PCR positive	ATT	–	Complete resolution	–
Kumar et al, 2011, India 26	9-year-old female	LJ/na/na	y	n	na	y/na/na/na	Montoux test positive (12 mm), chest X-ray and CT were normal	Surgical drainage with excision of coronoid process ATT started after HPE	F/s/o tuberculous osteomyelitis	Complete resolution, no recurrence	–
Upadhyay et al, 2011, India 3	14-year-old female	RPA/na/2 months	na	n	na	y/na/y/na	–	ATT	Epithelioid cell granulomas with central caseous necrosis with Langerhans giant cells	Complete resolution, no recurrence	–
Karjodkar et al, 2012, India 15	45-year-old male	RJ/y/2 months	na	y	na	y/na/y/na	Montoux positive PCR positive	ATT	–	Resolution and bone healing	–
Bai and Sun, 2014, China 27	31-year-old male	B/L J/y/2 months	y	na	n/y	y/na/na/na	ESR = 28 mm/hr, tuberculin test 21 mm PCR positive	Curettage of fistula and did PCR on yellowish white secretions ATT given	–	Resolution	–
Koul et al, 2014, India 28	16-year-old female	LPA/y/6 months	na	na	y/n	y/na/y/y	ESR = 45 mm/hr	ATT	–	Resolution	–
Gupta et al, 2016, India 29	66-year-old male	LPA/y/1 year	y	na	y/n	y/na/y/na	Raised ESR, tuberculin test positive 24 mm in first 48 hrs	ATT	–	No recurrence	–
Sambyal et al, 2016, India 14	3-year-old female	RJ/n/2 months	y	na	n/y	y/y/y/y	S-100, CD1A, PCR for TB neg ESR = 47 mm/hr, tuberculin test positive	ATT	Osteomyelitis	Resolution of lesion, no recurrence	–
Dalmia et al, 2016, India 30	21-year-old female	RPA/4 to 5 days	na	n	na	na/na/na/na	ESR normal, AFB neg TB PCR positive	ATT	Osteomyelitis of right hemi mandible with masseteric abscess	Complete resolution	–
Towdur et al, 2017, India 31	49-year-old female	LPA/y	na	na	na	na/na/y/na	Blood ix, HIV, Montoux and chest X-ray neg	Condylectomy with curettage	F/s/o tuberculous	–	–
Kalaiarasi et al, 2018, India 32	14-year-old male	RPA/na/na	y	n	y/n	y/na/na/na	ESR = 60 mm/hr, triple H negative, chest X-ray normal, AFB culture neg, sputum AFB neg	ATT 6 months	–	Complete resolution	–
Elzouiti et al, 2021, Morocco 6	50-year-old female	LJ/na/2 years	y	na	na	y/y/na/na	Chest X-ray normal	Left hemimandibulectomy	Ameloblastoma with mandibular and lymph node tuberculosis	Resolution of lesion, no recurrence	Simultaneous ameloblastoma with tuberculosis
Prashant et al, 2022, India 33	14-year-old female	RPA/y/2 months	n	na	na	y/na/y/na	GeneExpert TB, culture positive	Sequestrectomy, curettage of necrotic tissue in right condylar region	TB	–	–
Gupta et al, 2022, India 34	19-year-old female	RJ/n/2 months	na	n	na	y/na/na/y	Raised ESR, Montoux neg, chest CT normal, sputum AFB neg	Exploration, decortication	Suggestive of tuberculosis	Complete resolution	–

Abbreviations: AFB, acid fast bacilli; ATT, antituberculosis therapy; B/L J, bilateral jaw; ESR, erythrocyte sedimentation rate; HPE, histopathological examination; hr, hour; LJ, left jaw; LPA, left preauricular area; mm, millimeter; MRI, magnetic resonance imaging; n, no; na, not mentioned in publication; neg, negative; PCR, polymerase chain reaction; RJ, right jaw; RPA, right preauricular area; TB, tuberculosis; TMJ, temporomandibular joint; y, yes; f/s/o, features suggestive of.

### Conclusion

We have shared our experience of incidental detection of primary mandibular TB in a patient presenting with ameloblastoma-like picture. Clinical and radiological presentation of primary mandibular TB is not specific, making it a diagnostic challenge for clinicians. The histopathologic analysis is diagnostic for tuberculous osteomyelitis. It should form a part of differential diagnoses in endemic areas. If biopsy reveals tuberculous osteomyelitis, ATT can avoid the need of surgical intervention.
